# Comparison of the Quality of Discharge Letters Written by Large Language Models and Junior Clinicians: Single-Blinded Study

**DOI:** 10.2196/57721

**Published:** 2024-07-24

**Authors:** Joshua Yi Min Tung, Sunil Ravinder Gill, Gerald Gui Ren Sng, Daniel Yan Zheng Lim, Yuhe Ke, Ting Fang Tan, Liyuan Jin, Kabilan Elangovan, Jasmine Chiat Ling Ong, Hairil Rizal Abdullah, Daniel Shu Wei Ting, Tsung Wen Chong

**Affiliations:** 1 Department of Urology Singapore General Hospital Singapore Singapore; 2 Data Science and Artificial Intelligence Laboratory Singapore General Hospital Singapore Singapore; 3 Department of Endocrinology Singapore General Hospital Singapore Singapore; 4 Department of Gastroenterology and Hepatology Singapore General Hospital Singapore Singapore; 5 Department of Anesthesiology Singapore General Hospital Singapore Singapore; 6 Singapore Eye Research Institute Singapore National Eye Center Singapore Singapore; 7 Duke-NUS Medical School Singapore Singapore; 8 Artificial Intelligence Office Singapore Health Services Singapore Singapore; 9 Division of Pharmacy Singapore General Hospital Singapore Singapore

**Keywords:** artificial intelligence, AI, discharge summaries, continuity of care, large language model, LLM, junior clinician, letter writing, single-blinded, ChatGPT, urology, primary care, fictional electronic record, consultation note, referral letter, simulated environment

## Abstract

**Background:**

Discharge letters are a critical component in the continuity of care between specialists and primary care providers. However, these letters are time-consuming to write, underprioritized in comparison to direct clinical care, and are often tasked to junior doctors. Prior studies assessing the quality of discharge summaries written for inpatient hospital admissions show inadequacies in many domains. Large language models such as GPT have the ability to summarize large volumes of unstructured free text such as electronic medical records and have the potential to automate such tasks, providing time savings and consistency in quality.

**Objective:**

The aim of this study was to assess the performance of GPT-4 in generating discharge letters written from urology specialist outpatient clinics to primary care providers and to compare their quality against letters written by junior clinicians.

**Methods:**

Fictional electronic records were written by physicians simulating 5 common urology outpatient cases with long-term follow-up. Records comprised simulated consultation notes, referral letters and replies, and relevant discharge summaries from inpatient admissions. GPT-4 was tasked to write discharge letters for these cases with a specified target audience of primary care providers who would be continuing the patient’s care. Prompts were written for safety, content, and style. Concurrently, junior clinicians were provided with the same case records and instructional prompts. GPT-4 output was assessed for instances of hallucination. A blinded panel of primary care physicians then evaluated the letters using a standardized questionnaire tool.

**Results:**

GPT-4 outperformed human counterparts in information provision (mean 4.32, SD 0.95 vs 3.70, SD 1.27; *P*=.03) and had no instances of hallucination. There were no statistically significant differences in the mean clarity (4.16, SD 0.95 vs 3.68, SD 1.24; *P*=.12), collegiality (4.36, SD 1.00 vs 3.84, SD 1.22; *P*=.05), conciseness (3.60, SD 1.12 vs 3.64, SD 1.27; *P*=.71), follow-up recommendations (4.16, SD 1.03 vs 3.72, SD 1.13; *P*=.08), and overall satisfaction (3.96, SD 1.14 vs 3.62, SD 1.34; *P*=.36) between the letters generated by GPT-4 and humans, respectively.

**Conclusions:**

Discharge letters written by GPT-4 had equivalent quality to those written by junior clinicians, without any hallucinations. This study provides a proof of concept that large language models can be useful and safe tools in clinical documentation.

## Introduction

Large language models (LLMs) are artificial intelligence (AI) constructs with the capability to parse and generate human-like text based on pretraining with a vast corpus of unstructured text data. LLMs have shown promise in parsing large corpi of text data and generating human-like text across various domains, including health care [1].

One of the most promising applications of LLMs in health care is for automating tasks traditionally performed by health care professionals, such as writing discharge summaries and letters [2]. Despite the fact that such documents serve as a critical communication tool between hospital specialists and primary care providers (PCPs) in ensuring continuity of care, they are time-consuming to write, are often tasked to junior doctors, and are underprioritized when balanced against direct clinical care [3,4].

Prior studies have shown that inpatient hospital discharge summary quality is inadequate in many of the domains/elements required by The Joint Commission and endorsed at the Transitions of Care Consensus Conference (TOCCC) [3]. Many elements, including the provision of follow-up care plans, are of particular importance to patients being discharged to primary care. Improving discharge summary timeliness was shown to have positive effects on the quality of content and the transmission rate to PCPs [5,6].

The automation of inpatient discharge summaries has previously been explored using electronic discharge summary (EDS) programs. Maslove et al [7] showed no differences in PCP-reported overall quality between summaries generated by an EDS program and conventional dictated summaries. However, these studies assessed communication between inpatient and outpatient providers for single episodes of hospital admissions rather than summative information regarding a patient’s clinical condition across a longer follow-up, as would be the case with specialist outpatient care.

Moreover, the applications of LLMs in outpatient clinical documentation remain largely unexplored. Unlike inpatient admissions, which are typically self-contained and time-limited, outpatient specialist care can have more complex clinical documentation with longitudinal health records that may span across years. It remains to be seen if LLMs are able to parse clinical information with a strong temporal dimension and produce high-quality output.

There is currently no consensus on the single best way to objectively evaluate clinically relevant LLM output. However, approaches comparing LLM output against that generated by clinicians can help with assessment of a benchmark of “human-like” or “clinician-like” performance, which have been described in the related literature [8]. In addition, independent expert evaluation of LLM output has also been used for physician evaluation of LLM output [9]. We sought to combine both approaches in this study.

The aims of this study were to (1) evaluate the feasibility and quality of an LLM in writing discharge letters for patients discharged from a specialist outpatient clinic and (2) compare the discharge letters generated against similar letters written by human junior clinicians.

## Methods

### Ethical Considerations

This study was conducted in a simulated environment using only fictional patient data. As the use of fictional data does not fall under local Human Biomedical Research Act regulations [10], ethical approval was not required.

### Data Generation

Fictional patient data were generated by clinicians from the Singapore General Hospital’s Department of Urology, mimicking typical electronic medical records (EMRs) of 5 common patient groups seen at the outpatient clinic in a tertiary center. The time horizon for follow-up ranged from 6 months to 6 years. Data included initial referral letters from PCPs or emergency departments, initial and follow-up urology consultation notes, referrals to other departments, correspondence notes, and discharge summaries from relevant admissions. Information on the fictional cases is presented in Table 1. Each case varied in complexity, ranging between 4 and 10 documents, and included 1238-3009 characters (424-1110 tokens).

**Table 1:** Fictional case-mix data used for assessment.

Simulated case	Initial referral	Clinical condition	Follow-up duration	Additional/incidental clinical information
1	Elevated PSA^a^	Benign prostatic hyperplasia	6 years	Initially declined biopsy and opted for PSA monitoring, but ultimately underwent a transperineal prostate biopsy and developed acute retention of urine postoperatively
2	LUTS^b^	Overactive bladder	3 years	Failed behavioral modification measures and experienced adverse effects from multiple lines of anticholinergic medications
3	Asymptomatic microhematuria	Renal calculus	1.5 years	Incidental pancreatic lesion on imaging and was referred to general surgery for further evaluation
4	Ureteric colic	Distal ureteric stone	6 months	Failed medical expulsive therapy and underwent ureteroscopy and lithotripsy; also noted to have hypertension perioperatively
5	Erectile dysfunction	Erectile dysfunction	2.5 years	Noted to have abnormal ECG^c^ and was referred to cardiology for further assessment prior to initiation of PDE-5^d^ inhibitors

^a^PSA: prostate-specific antigen.

^b^LUTS: lower urinary tract symptoms.

^c^ECG: electrocardiography.

^d^PDE-5: phosphodiesterase-5.

### Discharge Letter Generation

GPT-4 is a state-of-the-art LLM developed by OpenAI with a parameter count of 1.76 trillion, and has exhibited human-level performance on professional and academic benchmarks [11]. GPT-4, with an 8000-token context length, was provided with the fictional EMRs and tasked to write discharge letters to PCPs. The prompt instructed GPT-4 to assume the role of a physician assistant in a urology clinic, specifying the context of the task (“this patient is being discharged”) and the target audience (“meant to be read by the general practitioner”). To reduce the risk of hallucination, prompts for safety included specific instructions to include only information provided in the fictional EMR [12]. Desired content guidelines were provided to standardize the structure of the discharge letter. Prompts for style included instructions to write in prose, in a cordial and concise manner, and not to exceed half a page of text unless necessary. To evaluate the generative capacity of the LLM, only the above instructions (but no examples) were provided to GPT-4, which is a technique known as “zero-shot” prompting [13].

Three separate discharge letters were generated for each fictional case. One was generated by GPT-4 and the comparators were two letters that were written concurrently by junior clinicians from the Department of Urology using the same set of fictional patients. For the comparator letters, use of generative AI tools or other automated summarization methods was disallowed.

Full prompt instructions are shown in Multimedia Appendix 1. Sample medical records and discharge letters are shown in Multimedia Appendix 2.

### Comparison of Discharge Letters

All letters underwent an initial independent screening by two study team members (JYMT and SRG) for factual inaccuracy and grammatical errors. For letters generated by GPT-4, this included screening for instances of hallucination by the language model. For cases 3 and 4, letters were also assessed with respect to their attention to secondary problems beyond the prompted surgical issue based on the presence or absence of follow-up recommendations for other medical issues found during the course of the patient’s treatment.

**Figure 1:**
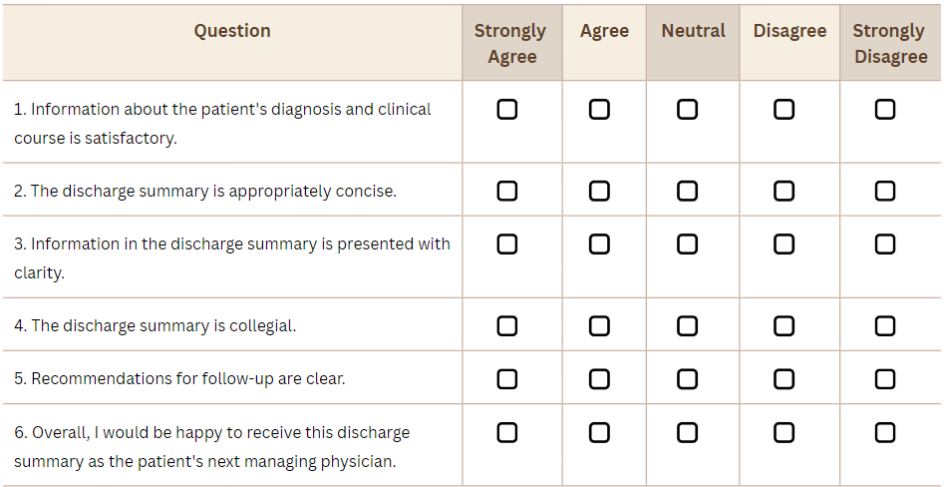
Scoring questionnaire.

A panel of 5 senior primary care physicians was presented with the discharge letters written by GPT-4 and junior clinicians in a blinded fashion. The participating physicians on the expert panel had an average clinical practice experience of 34.4 years. They compared the letters produced by GPT-4 and junior clinicians using a standardized rubric, but they were not informed that one of the letters was LLM-generated. Letters were evaluated on a 5-point Likert scale for completeness of information, conciseness, clarity, collegiality, whether follow-up care plans were articulated, and a single overall satisfaction question (Figure 1). Since there are no validated tools for assessment of outpatient discharge letter quality, questionnaire items were selected based on elements endorsed by the TOCCC for inpatient summaries.

### Data Analysis

SPSS version 26.0 (IBM Corp) was used for the statistical analysis of quantitative data. Total scores and arithmetic means with SDs were calculated for all responses as a whole and for responses to each individual item. As the data were nonnormally distributed (Shapiro-Wilk test *P*<.001), differences in means were compared using the Mann-Whitney *U* test.

## Results

Discharged letters generated by GPT-4 averaged 276 words (1462 characters), in comparison to 155 words (737 characters) for letters written by junior clinicians. No instances of hallucination and no grammatical errors were present in the letters generated by GPT-4. While no factual inaccuracies were found in the letters written by the junior clinicians, they made an average of 1.7 grammatical errors per letter.

Each panel member scored all 15 letters, for a total of 75 responses. The mean total score per letter was 22.99 (SD 6.26). GPT-4 scored higher on question items 1 (information), 3 (clarity), 4 (collegiality), 5 (follow-up plans), and 6 (overall satisfaction), and received higher total mean scores as compared to those of human subjects, although this difference was only statistically significant for item 1. GPT-4 received lower scores on item 2 (conciseness). The complete results are shown in Table 2.

**Table 2:** Comparison of mean scores for each evaluation item of discharge letters written by GPT-4 and junior clinicians.

Item	GPT-4 (n=25), mean (SD)	Junior clinicians (n=50), mean (SD)	*P* value
Information	4.32 (0.95)	3.70 (1.27)	.03
Conciseness	3.60 (1.12)	3.64 (1.27)	.71
Clarity	4.16 (0.94)	3.68 (1.24)	.12
Collegiality	4.36 (1.00)	3.84 (1.22)	.05
Recommendations	4.16 (1.03)	3.72 (1.13)	.08
Overall satisfaction	3.96 (1.14)	3.62 (1.34)	.36
Total	24.56 (5.13)	22.20 (6.66)	.20

GPT-4 also had some unanticipated strengths when compared against the letters written by the junior clinicians. In particular, GPT-4 demonstrated attention to secondary problems that were not explicitly included in the original prompt. In cases 3 and 4, GPT-4 provided follow-up recommendations based on the given fictional records for the incidental pancreatic lesion (“we recommend annual imaging studies to monitor the renal stone and the pancreatic lesion, in collaboration with the General Surgery team”) and perioperative hypertension (“he is recommended to continue with Amlodipine 5mg daily for his hypertension”). In contrast, although the junior clinicians also made mention of the pancreatic lesion and subsequent general surgery referral, they did not provide follow-up recommendations to the PCP.

## Discussion

### Principal Findings

To our knowledge, this study represents the first blinded comparison of discharge letters written by an advanced LLM versus human physicians.

Our analysis showed that GPT-4 performed comparably against junior clinicians from an inpatient specialist department, with equivalent scores in clarity, collegiality, and ability to articulate follow-up recommendations. GPT-4 also demonstrated the ability to generate a holistic patient summary with attention to secondary medical issues and made fewer grammatical errors than human comparators. The higher score in information provision highlights the capability of language models with strong embeddings to capture and present important details from patient EMRs. However, this appears to have come at the expense of a relative lack of conciseness, as indicated by GPT-4 receiving a lower score in this domain. More specific metaprompting with instructions for word limits could overcome this limitation. Nevertheless, the importance of an LLM’s ability to rapidly digest and summarize a large free-text corpus cannot be overstated, which offers the potential for more consistent information relay between health care providers.

These findings echo prior work in the field comparing EDSs against traditional dictated summaries. While earlier digitization efforts were limited by technological capabilities of the era, advances in natural language processing and linguistic ability allow modern language models to write fluently and mimic human prose convincingly. While the potential of language models in this area of health care has been discussed [2], these first results prove that LLMs do have the capability to automate time-consuming tasks for health care professionals. This is a transformative opportunity that could be a useful tool in bridging communications between PCPs and inpatient specialists by ensuring the quality and accuracy of discharge letters. Clinicians burdened with documentation in addition to clinical duties stand to benefit immensely from automation of these tasks, allowing more time for patient contact and clinical care. Likewise, patients could benefit from consistent, timely handovers and a more robust system of continuity of care, instead of the vagaries of the junior clinicians writing their discharge letters. With appropriate refinement of prompt instructions, we postulate that this transformative benefit can extend beyond automating the documentation work of physicians to other health care professionals (eg, nurses and pharmacists).

### Limitations and Prospects

The exclusive reliance on fictional patient data in this study, while necessary for ethical and patient data considerations, might not capture the real-world complexities in patient EMRs. We attempted to overcome this limitation by having the fictional EMRs written by physicians who also regularly see patients in the urology outpatient clinic on a day-to-day basis and enter clinical notes into actual patient EMRs. The small number of panel assessors as well as the use of clinical cases from a single surgical specialty may reduce the generalizability of these findings. No evaluation was performed as to whether the panel assessors inferred that any of the letters were LLM-generated. The time taken for junior clinicians to write discharge letters was not measured, precluding comparison in terms of time savings against an LLM. In addition, despite the advantages of LLM tools, misinformation phenomena such as hallucination and concept drift remain areas of concern. We propose that such an AI tool should be used to augment, not replace, human-written discharge letters, and that physician oversight should still be required before discharge documents are handed to patients. Users of LLMs as productivity aids in clinical medicine must also be aware of prevailing privacy protection policies, and closed-access LLM implementations (eg, on a private server) may be needed for regulatory compliance. While automating discharge letter writing may undermine the clinical reasoning process that physicians undertake [14], we believe that LLMs implemented with a “human in the loop” can eventually exert a long-term training effect, reinforcing these cognitive skill sets [15].

Future research using actual patient EMRs will be necessary to confirm these preliminary findings. Studies may be expanded to care across different medical specialties to ascertain the ability of language models to comprehend abbreviations and concepts from different disciplines. Fine-tuning a language model on medical information and patient EMRs and refining metaprompts are avenues to further improve the quality of discharge letters generated by LLMs and to balance conciseness with information density.

### Conclusion

The emergence of AI in health care promises a paradigm shift in the way clinical medicine is practiced. Our study provides insight into AI’s capacity to optimize the continuity of patient care. As evaluation of language models for clinical applications continues to advance, we propose benchmarking their performance against human counterparts to determine feasibility and assess output quality.

## Appendix

Multimedia Appendix 1 Prompt instructions.

Multimedia Appendix 2 Sample fictional clinical records and discharge letters.

## Abbreviations

AI: artificial intelligence
EDS: electronic discharge summary
EMR: electronic medical record
LLM: large language model
PCP: primary care provider
TOCCC: Transitions of Care Consensus Conference

## Data Availability

The data sets generated and/or analyzed during this study are available from the corresponding author on reasonable request.
