# Supplementary figures and images for: Comparison of the Quality of Discharge Letters Written by Large Language Models and Junior Clinicians: Single-Blinded Study
Source: J Med Internet Res. 2024 Jul 24;26:e57721. doi: 10.2196/57721 (PMC11306941; doi:10.2196/57721)

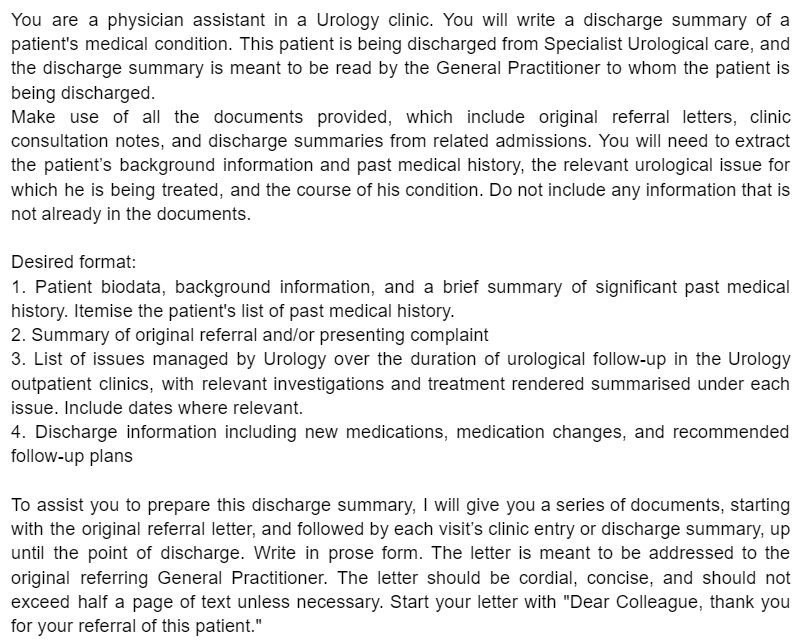

Supplement: Multimedia Appendix 1 [file jmir_v26i1e57721_app1.png]
