# Supplementary material for: Comparison of the Quality of Discharge Letters Written by Large Language Models and Junior Clinicians: Single-Blinded Study
Source: J Med Internet Res. 2024 Jul 24;26:e57721. doi: 10.2196/57721 (PMC11306941; doi:10.2196/57721)
Supplement: Multimedia Appendix 2 [file jmir_v26i1e57721_app2.docx]

Sample fictional clinical records and discharge letters

| **Sample consultation notes from Case 1** | **GPT-4** | **Medical Officer 1** | **Medical Officer 2** |
| --- | --- | --- | --- |
| Uro Consult 8 June 2018  65/M  NKDA  Non-smoker  Nil PMH  Referred for elevated PSA 5.3 Feb 2018.  Also c/o voiding LUTS - poor stream, hesitancy.  No frequency/urgency.  No GH/dysuria  No aggravating factors.  No fhx of renal/prostate/bladder CA  OE  Abdo soft, non-tender  Penis, scrotum, testes NAD  DRE - benign, 3FB  BSUS  b/l no HN  PV 45cc IPPG1  Offered further evaluation of elevated PSA with MRI prostate - not keen, prefers to trend PSA. Understands risk of missed CA diagnosis.  P:  UFEME, Urine c/s  Alfuzosin - counseled re: giddiness  TCU 3/12 UFRU PSA OA  Uro Consult 5 Jan 2020  MRI Prostate Dec 2019: PIRADS 3 x1 0.4cm Left Anterior PZ, Prostate volume 75cc.  Offered biobot prostate biopsy - keen. Counselled re: risks of bleeding, infection, ARU.  P:  List for biobot prostate biopsy on 17 Feb 2020 as DS under GA  PAAC  Urine c/s  PP  Uro Consult 8 Mar 2023  Well  Happy w LUTS  UF 12/450 RU 13ml  PSA 1.25 (x2=2.5)  Patient request for transfer of care to OPS as he feels his condition is stable.  P:  Open date Uro  Cont. alfuzosin and finasteride  Memo to OPS to continue BPH medications | Dear Colleague,  Thank you for your referral of Mr. Wong Chin Jin, a 65-year-old gentleman, who initially presented to our Urology service with elevated Prostate-Specific Antigen (PSA) levels and lower urinary tract symptoms.  Over the course of five years, we closely monitored Mr. Wong's PSA levels and urinary symptoms. Initially, he was hesitant about further evaluation of his elevated PSA levels, opting instead for symptomatic management and PSA trending. We instituted Alfuzosin, and he reported satisfaction with the management of his symptoms.  In 2019, Mr. Wong consented to an MRI of the prostate. The results showed a PIRADS 3 x1 0.4cm lesion in the Left Anterior Peripheral Zone. Consequently, he underwent a Transperineal Biobot Prostate Biopsy in February 2020, which unfortunately led to a post-operative complication of Acute Retention of Urine (ARU). However, he recovered well, and the histopathological examination confirmed the presence of Benign Prostatic Hyperplasia (BPH).  From March 2020, Mr. Wong was started on Finasteride, in addition to the ongoing Alfuzosin, to manage his BPH. Subsequent follow-ups demonstrated satisfaction with his symptom control, and his PSA levels notably decreased to around 2.5.  As of his last consultation in March 2023, Mr. Wong feels his condition is stable and has requested to be transferred to your care in the Outpatient Department. We recommend that he continues his current medications of Alfuzosin and Finasteride for BPH. We have left an open date for him in Urology, should he require any specialized care in the future.  Thank you for entrusting us with Mr. Wong's urological care.  Sincerely,  [Your Name]  [Your Position] | Dear Senior Colleague,  Thank you for your referral for Mr Wong who is known to Uro for BPH stable on alfuzosin and finasteride, currenlty keen for f/u in your clinic setting. He was initially referred for Raised PSA of 5.3 with the following management.  65/M  NKDA  Non-smoker  Nil PMH  Uro Issues  1. Raised PSA  - PSA: 5.3 (Feb 2018) > 5.5 (Sept 2018) > 5.9 (Sept 2019) > PSA 1.30x2=2.6 (4 Mar 2022) > PSA 1.25x2=2.5 (8 Mar 2023)  - Dec 2019: MRI Prostate - PIRADS 3 x1 0.4cm Left Anterior PZ, Prostate volume 75cc.  - 17 Feb 2020 - Biobot Prostate Biopsy, Histo: BPH  2. LUTS 2' to BPH  - Dec 2019: MRI Prostate - PIRADS 3 x1 0.4cm Left Anterior PZ, Prostate volume 75cc  - 8 June 2018 - Started on alfuzosin  - 17 Mar 2020 - started on finasteride  - 8 March 2023: UF 12/450 RU 13ml  We would appreciate you continued care of this patient and to prescribe his medications as required. He has been given an open date from Urology. Thank you for your continued care of this patient.  Regards | Dear Colleague, thank you for your referral of this patient with elevated PSA 5.3 done as part of health screening and was diagnosed to have BPH. He was initially started on alfuzosin for his associated voiding LUTs and declined MRI prostate for further evaluation from June 2018 to Sep 2019. His PSA trend during the corresponding period was 5.3 > 5.5 > 5.9.  He was eventually agreeable for MRI prostate which showed PIRADS 3 x1 0.4cm Left Anterior PZ, prostate volume 75cc. Transperineal biobot biopsy was eventually done on 17/2/20 which showed BPH. Finasteride was added on top of alfuzosin in March 2020 with improvement in his LUTs. His condition has since has been stable from 2020 to 2023 with the following PSA trend: 2.5 > 2.6 > 2.5. He eventually requested for transfer of care to OPS in view of his stable condition.  Kindly assist in following up for his BPH and continue his alfuzosin and finasteride.  Thank you. |
| **Sample consultation notes from Case 4** | **GPT-4** | **Medical Officer 1** | **Medical Officer 2** |
| Uro Consult 28 Dec 2022  32/M  NKDA  Non-smoker  Works office desk job (IT)  Nil PMH  R ureteric colic  Seen A&E 2 weeks ago  Still having pain intermittently  Loin to groin, colicky  One episode gross hematuria which resolved  No dysuria  No fever  No prior stone hx  No fmhx cancer/stones  A&E UFEME 40/10/0  KUB XR ?right ureteric stone  OE  Abdo soft, non-tender  Renal punch neg  Penis, scrotum, testes NAD  DRE - benign, 2.5FB  P:  UFEME, urine culture  CT IVP  TCU 2/52 after  Discharge Summary  Patient Name: Aaron Lee  Admission Date: 6 March 2023  Discharge Date: 6 March 2023  Admission Type: Day Surgery  Elective admission for Right Ureteroscopy and laser lithotripsy.  Patient underwent Right Ureteroscopy and laser lithotripsy on 6 Mar 2023.  Peri-operatively was noted to have hypertension and started on amlodipine 5mg OM.  Discharged well and stable on 6 Mar 2023.  List for DJ stent removal on 18 Mar 2023 in Urology clinic.  TCU OPS in 1/12 to review hypertension  Discharge medications:  Amlodipine 5mg OM 1 month  Augmentin 1g BD 5 days  Paracetamol 1g QDS PRN 7 days  Uro Procedure 18 Mar 2023  Flex scope and R DJ stent removal performed.  Stent removed in entirety - tip intact.  P:  TCU 3/12 XR KUB OA  Uro Consult 28 June 2023  Well  XR KUB - no stone seen  Did not go to OPS for Hypertension follow up in March.  P:  Discharge to OPS - memo to followup HTN  Open date Uro  Return advice given | Dear Colleague,  Thank you for your referral of Mr. Aaron Lee, a 32-year-old IT professional with no prior medical history. He was initially seen in A&E for right-sided ureteric colic on 17 Dec 2022.  Mr. Lee presented with intermittent loin to groin pain, with one episode of gross hematuria, which later resolved. A subsequent CT IVP revealed a 3mm right distal ureteric stone. Given the persistence of the stone on follow-up imaging, despite an initial attempt at conservative management with Medical Expulsive Therapy (MET), he underwent a successful Right Ureteroscopy and laser lithotripsy on 6 Mar 2023.  During his day surgery admission for the procedure, he was noted to have hypertension and was started on Amlodipine 5mg once daily. A follow-up double J (DJ) stent removal was conducted uneventfully on 18 Mar 2023. A follow-up XR KUB on 28 June 2023 revealed no residual stones.  Upon discharge, he is recommended to continue with Amlodipine 5mg daily for his hypertension. He had failed to attend his scheduled follow-up at the Outpatient Services (OPS) for his hypertension in March. Thus, I would appreciate if this could be followed up in your care.  Please also advise him to return to Urology services if he experiences similar symptoms in the future. Thank you again for your referral.  Kind regards,  [Your Name]  [Your Title] | Dear Senior Colleague,  Thank you for your referral for Mr Lee who is known to Uro for Right Ureteric stone s/p Right Ureteroscopy and laser lithotripsy, currenlty keen for f/u in your clinic setting. He was also noted to have newly diagnosed HTN started on amlodipine 5mg OM. He was initially referred for Right Ureteric Colic with the following management.  32/M  NKDA  Non-smoker  Works office desk job (IT)  PMH  1. HTN - started on Amlodipine 5mg OM  Uro Issues  1. Right ureteric stone s/p Right Ureteroscopy and laser lithotripsy on 6 March 2023  - Initial presentation of Right ureteric colic on 28 Dec 2022  - DRE - benign, 2.5FB  - 15 Jan 2023: CT IVP: 3mm right distal ureteric stone  - s/p MET (Tamsulosin)  - 28 Feb 2023: CT KUB: 3mm right distal ureteric stone, no migration of stone. Mild right hydronephrosis seen  - s/p Right Ureteroscopy and laser lithotripsy on 6 March 2023 and flex cysto and stent removal on 18 March 2023  - 28 June 2023: XR KUB - no stone seen  Other issues  1.HTN  - started on Amlodipine 5mg OM on 6 Mar 2023  We would appreciate you continued care of this patient and to prescribe his medications as required. He has been given an open date from Urology. Thank you for your continued care of this patient.  Regards | Dear Colleague, thank you for your referral of this patient with ureteric colic. The following investigations were done:  Urine cultures negative  CT IVP: 3mm right distal ureteric stone  He was initially keen for Medical Expulsive Therapy and was given a 6 week course of tamsulosin from Jan 2023 to Feb 2023. A repeat CT KUB showed persistence of 3mm right distal uretic stone with no migration and newly developed right hydronephrosis. He was eventually keen for Right ureteroscopy and laser lithotripsy which was done on 6 March 2023 with removal of DJ stent (inserted intraoperatively) on 18 March 2023. A repeat XR KUB in June 2023 did not show presence of any stones.  He was also noted to have hypertension on the day of uretoscopy and was started on amlodipine 5mg OM. However he defaulted follow up with OPS for his hypertension.  Kindly assist in following up with his hypertension. Return advice has been given to patient.  Thank you! |
